# Zoledronate Bound to Ceramics Increases Ectopic Bone Volume Induced by rhBMP6 Delivered in Autologous Blood Coagulum in Rats

**DOI:** 10.3390/biomedicines9101487

**Published:** 2021-10-16

**Authors:** Nikola Stokovic, Natalia Ivanjko, Igor Erjavec, Anita Breski, Mihaela Peric, Slobodan Vukicevic

**Affiliations:** 1 Laboratory for Mineralized Tissues, Centre for Translational and Clinical Research, University of Zagreb School of Medicine, 10000 Zagreb, Croatia; nikola.stokovic@mef.hr (N.S.); natalia.ivanjko@mef.hr (N.I.); igor.erjavec@mef.hr (I.E.); 2 Department of Pathology and Cytology, University Hospital Centre Zagreb, 10000 Zagreb, Croatia; anita.breski@kbc-zagreb.hr; 3 Department for Intracellular Communication, Centre for Translational and Clinical Research, University of Zagreb School of Medicine, 10000 Zagreb, Croatia; mihaela.peric@mef.hr

**Keywords:** bone regeneration, bisphosphonates, zoledronate, bone morphogenetic proteins, BMP, BMP carrier, osteogrow

## Abstract

Autologous bone graft substitute (ABGS) containing rhBMP6 in autologous blood coagulum (ABC) with synthetic ceramics is a novel therapeutic solution for bone repair. The aim of this study was to investigate whether the application of Zoledronate (ZOL) with ABGS might enhance the properties of newly formed bone. The effect of ZOL on bone induction was tested in a rat subcutaneous implant model. ZOL bound to synthetic ceramics was added into ABGS implants, and the quantity, quality, and longevity of the induced bone were assessed by micro-CT, histomorphometry, and histology over a period of 365 days. Local use of ZOL in the ABGS implants with ceramics had no influence on the bone volume (BV) on day 14 but subsequently significantly increased BV on days 35, 50, 105, 140, and 365 compared to the control implants. Locally applied ZOL had a similar effect in all of the applied doses (2–20 µg), while its systemic use on stimulating the BV of newly induced bone by ABGS depended on the time of application. BV was increased when ZOL was applied systemically on day 14 but had no effect when applied on day 35. The administration of ZOL bound to ceramics in ABGS increased and maintained the BV over a period of one year, offering a novel bone tissue engineering strategy for treating bone defects and spinal fusions.

## 1. Introduction

Bone morphogenetic proteins (BMPs) are growth and differentiation factors known for their ability to induce cartilage and bone and have a morphogenic role in the development and maintenance of tissue structure throughout the body [1,2]. The ability of BMPs to induce bone was evaluated in various animal models used for the translation of the bone regeneration principle for clinical use [3,4,5,6]. Finally, the osteoinductive device containing rhBMP2 and bovine collagen carrier has been approved for use in anterior lumbar interbody fusion (ALIF) [7] and acute tibial fractures [8,9]. However, the off-label use of a rhBMP2 therapeutic in anterior or posterior cervical fusion, posterolateral lumbar fusion, and long bone non-unions has resulted in safety issues [10] that can be attributed to large rhBMP2 doses, the immunogenicity of animal-sourced collagen, and variable inferiority to the autologous iliac crest bone [11].

We have demonstrated that our novel autologous bone graft substitute (ABGS) containing rhBMP6 within autologous blood coagulum (ABC) with or without compression resistant matrix (CRM) restores large bone defects and achieves successful lumbar fusion in rabbits and sheep [2,12,13,14,15,16,17,18,19]. Importantly, newly induced bone successfully fused with native bone, achieving complete osseointegration, which can be defined as functional fusion between new and old bone [20]. BMP6 is more potent in promoting osteoblast differentiation *in vitro* and inducing bone regeneration *in vivo* than BMP2 due to resistance to Noggin inhibition and spanning across BMP type I and type II receptors [1,21,22]. In addition, ABC serves as a physiological biocompatible carrier [12], promoting the tight binding of rhBMP6 to plasma proteins within the fibrin meshwork. ABC further allows a sustained release of rhBMP6 and suppresses foreign body responses elicited by the mineral-rich ceramics used as CRM [2,13,14]. Furthermore, synthetic ceramics are more preferred CRM than allografts [16,17,23] due to the reduced risk of viral transmission and immunogenicity [24]. Furthermore, biphasic calcium phosphate contains beta-tricalcium phosphate (TCP) as a more resorbable component and more stable hydroxyapatite (HA) at various ratios, thus allowing the proper tuning of CRM resorbability [25]. The biology of BMP-induced bone formation has been studied at various time points in a rat subcutaneous bone formation assay [12,13,14,17], rabbit segmental bone defects, and in a rabbit and sheep posterolateral and anterior lumbar spinal fusion models [13,14,15,16]. In addition, ABGS has been proven to be safe and efficacious in patients with distal radial fractures (DRF; EudraCT 2014-005101-21) [26] and high tibial osteotomy (HTO; EudraCT 2015–001691-21) [27].

BMPs induce new bone formation by stimulating mesenchymal stem cell (MSCs) differentiation into chondroblasts and pre-osteoblasts [22]. However, they also induce osteoclast differentiation from hematopoietic stem cells and increase their activity directly and indirectly through the RANK-RANKL system [12]. Bisphosphonates prevent resorption and bone loss by mediating osteoclast apoptosis [28]. Therefore, the co-application of BMPs with bisphosphonates was previously proposed to achieve optimal bone regeneration [29,30,31,32,33,34,35,36,37,38,39,40] and was examined in a rat ectopic bone assay [29,30,31,32,33], rat bone conduction chamber model [39,40] as well as in rat critical-sized defect and fracture models [34,35,36,37,38]. Zoledronate (ZOL), nitrogenous heterocyclic bisphosphonate, is the most potent and long-acting bisphosphonates used in patients with osteoporosis, Paget’s disease, myeloma, and cancer to reduce adverse skeletal events [41].

The aim of the present study was to elucidate whether the application of ZOL might enhance the properties of bone induced by novel ABGS (rhBMP6/ABC) implants containing synthetic biphasic calcium phosphate ceramic particles. Therefore, we investigated the effects of ZOL on different phases of BMP-mediated bone induction and on the long-term maintenance of newly formed bone. Moreover, we evaluated ZOL effects with respect to the route and timing of the administration.

## 2. Material and Methods

### 2.1. Test Items

The test items included rhBMP6 produced by Genera Research (Zagreb, Croatia) [13,17], biphasic synthetic ceramic particles produced by CaP Biomaterials (East Troy, WI, USA) [17], and zoledronate (Teva, Petah Tikva, Israel). Tested synthetic ceramic particles contained beta-tricalcium phosphate (TCP) and hydroxyapatite (HA) in an 80/20 ratio, with particle sizes ranging from 500 µm to 1700 µm. Ceramic particles were produced according to the previously described procedure [17]. In brief, the material was produced by reacting calcium hydroxide and phosphoric acid to form a slurry that was mixed with viscosity modifiers and hydrogen peroxide. While the slurry dried, peroxide decomposed, and liberated oxygen caused the formation of pores. The dry foam was sintered, ground, and sieved to make granulate in 500–1700 µm size range. Optical imaging (Wild Photomakroscope M400) was used to determine the average pore size, while the porosity was determined from the measured bulk density as a percentage of the theoretical density. The average pore diameter was 175 µm, while the total porous volume was 86%.

### 2.2. Experimental Design

To elucidate the effect of ZOL on ectopic osteogenesis, we conducted several experiments using the rat subcutaneous implant assay. It is a well established rodent model that is used for investigating the biology of bone formation and for testing novel osteoinductive therapies with high translatability to higher order animals and humans [42]. First, we investigated whether the local application of ZOL (10 µg) bound to the ceramics in ABGS (rhBMP6/ABC) implants affected bone formation in a rat subcutaneous assay on day 14. Next, we explored how local ZOL dosing in ABGS implants affected new bone formation at 35 and 50 days following implantation and at a long-term follow up at days 140 and 365 regarding the bone volume and structure compared to control implants. To test whether the ZOL effect could be reproduced via systemic administration, ZOL was applied either locally in the implant or as a single intravenous dose applied on days 14, 21, or 35 following implantation. The follow-up period was 50 and 105 days. The number of specimens per group was 5–6 and was determined based on previously published studies using this model [5,42].

### 2.3. Experimental Animals

Sprague Dawley laboratory rats (lat. *Rattus norvegicus*, male, 8–12 weeks old, body weight around 250–350 g) were used in all of the experiments. Animals were bred and housed at the Laboratory for Mineralized Tissues (University of Zagreb School of Medicine, Zagreb, Croatia; HR-POK-001). The temperature in the facility was 18–24 °C with relative humidity of 40–70%. In the animal facility there was 12/12 h light/dark cycle per day, and the noise level was 60 dB. A standard GLP diet and fresh water were provided *ad libitum*.

Animal care complied with SOPs of the animal facility as well as with the European conventions for the Protection of Vertebrate Animals used for Experimental and other Scientific Purposes (ETS 123). The ethical principles of the study ensured compliance with European Directive 010/63/E, the Law on Amendments to Animal Protection Act (Official Gazette 37/13, the Animal Protection Act (Official Gazette 102/17), the Ordinance on the Protection of Animals Used for Scientific Purposes (Official Gazette 55/13), and FELASA recommendations, and evaluation of the Ethics Committee at School of Medicine, University of Zagreb and the National Ethics Committee (EP 191/2019 and EP 296/2020).

### 2.4. Implant Preparation

Autologous blood samples (0.5 mL) were collected in tubes without an anticoagulant substance, and rhBMP6 (20 μg) was immediately mixed with blood, and the mixture was drawn into a sterile syringe either with ceramic particles (100 mg) or without them, and the blood was left to coagulate. In implants with ZOL, ceramic particles were soaked for 2 h with a solution containing ZOL (2–20 µg according to the experimental design) in a volume of 100 µL before being mixed with blood. All implants were implanted 60 min following preparation.

### 2.5. Rat Subcutaneous Implant Assay

Rats were anaesthetized by a combination of xylazine 5 mg/kg (i.m.) and ketamine 100 mg/kg (s.c.). A vertical skin incision was made under a sterile condition in the median line over the thoracic region. Implants were inserted bilaterally into subcutaneous pockets in the axillary region as described [13,14,17]. Rats were euthanized at specified time points, and the implants were surgically extracted from the axillary region and were processed for histology, histomorphometry, and microCT analyses.

### 2.6. Histology

Extracted implants were fixed in 4% formalin for ten days and were decalcified using 14% EDTA in 4% formalin solution for 20 days. They were then paraffin-embedded and cut at 6 μm slice thickness as described [14]. Selected implants were processed undecalcified, as previously described [16], and were cut at 35 µm (ground sections). Histological sections were stained by modified Goldner, Von Kossa, Hematoxylin-Eosin (HE), or Sanderson’s Rapid Bone Stain with Van Gieson picrofuchsin [15,16].

### 2.7. Histomorphometry

Histomorphometric analyses were conducted to evaluate the effect of ZOL on the number of osteoclasts, bone microarchitecture, and the amount of bone and bone/bone marrow ratio as previously described [16,17,18]. Briefly, osteoclasts were localized by histochemical acid phosphatase detection on HE-stained sections and were counted in five fields (one field = 0.4 mm^2^) on each section. The bone structure was analyzed on histological sections stained by modified Goldner’s stain and were imaged at 10× (1.83 pixel/μm) magnification using an Olympus BX53 Upright Microscope equipped with a DP27 camera (5 megapixels, 15 fps) and operated by cellSens Dimension software (Olympus, Tokyo, Japan). Image analysis was conducted using Photoshop software (Adobe Systems, San Jose, CA, USA) and Fiji ImageJ software (version 1.51r; NIH, Bethesda, MD, USA). Results were expressed as bone/bone marrow (b/bm) ratio.

### 2.8. MicroCT Analyses

To analyze the effect of ZOL on the amount and structure of newly formed bone, extracted implants were scanned using a 1076 SkyScan µCT device (Bruker SkyScan, Billerica, MA, USA). Scanning parameters were set as follows: resolution 18 µm, frame averaging 2, 0.5 mm aluminum filter, and rotation step 0.5° [43]. Acquired images were reconstructed using NRecon software (Bruker SkyScan, Billerica, MA, USA). Following image reconstruction, CTAn software (Bruker SkyScan, Billerica, MA, USA) was used to calculate bone and ceramics volume as well as trabecular parameters (trabecular thickness and trabecular number) among experimental groups [17].

### 2.9. Data Analysis

Distribution of numerical data sets was tested with the Kolmogorov–Smirnov test. Depending on the distribution, experiments with two experimental groups were analyzed using unpaired *t*-tests or the Mann–Whitney test. For multiple group comparisons, one-way ANOVA with Tukey multiple comparisons or the Kruskal—Wallis test with Dunn’s multiple comparisons test were used. Data are shown as mean with standard deviation (SD) or as median with all values (dots) including minimum and maximum. All *P* values below 0.05 were considered significant and are marked with asterisks as follows: * (*P* ≤ 0.05), ** (*P* ≤ 0.01), *** (*P* ≤ 0.001). Statistical software GraphPad Prism (v.8.4.3.) was used for all statistical calculations.

## 3. Results

### 3.1. Zoledronate Local Short-Term Effect on Bone Induction

**Figure 1:**
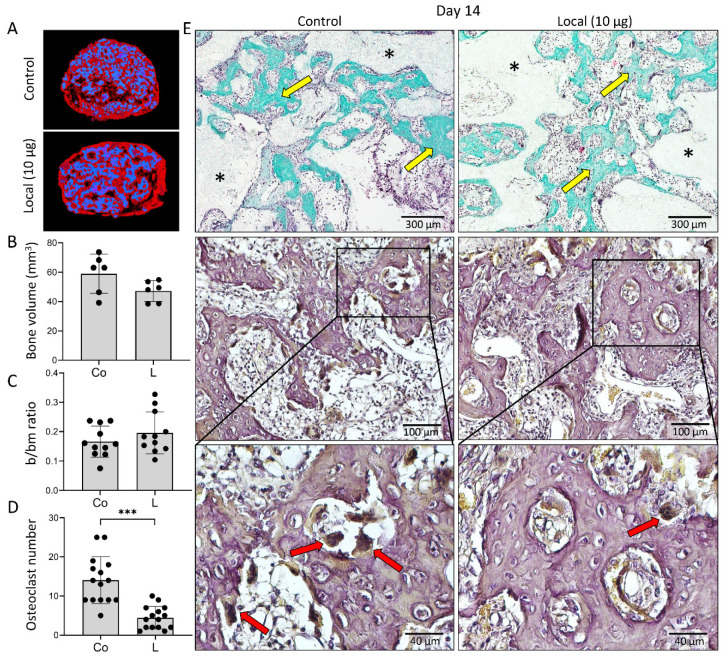
**Addition of zoledronate (ZOL) to ABGS (rhBMP6/ABC) implants with ceramics had no effect on the bone formation 14 days after subcutaneous implantation but decreased the number of osteoclasts.** (**A**) Newly formed bone (red) and ceramics (blue) are shown on 3D reconstruction slices among experimental groups. (**B**) MicroCT determined bone volume (mm^3^) and (**C**) histomorphometrically determined bone/bone marrow (b/bm) ratio among control (Co) and locally added ZOL (L) implants. (**D**) Number of osteoclasts counted on histological sections. (**E**) Histological sections of BCS induced by implants with and without ZOL. Sections were stained with Goldner where newly formed bone is marked with yellow arrow, while black asterisks indicate ceramic particles (1st row). Osteoclasts (red arrows) were localized on HE-stained sections using histochemical detection of TRAP (2nd and 3rd row). Scale bars are shown in the lower right corner. All *P* values below 0.05 were considered significant and are marked with asterisks as follows: * (*p* ≤ 0.05), ** (*p* ≤ 0.01), *** (*p* ≤ 0.001).

ABGS (rhBMP6/ABC) implants containing ceramics with and without ZOL induced new bone formation on day 14. The newly formed bone and ceramic particles formed a bone–ceramic structure (BCS) [17] consisting of a cortical bone at the BCS boundaries, bone on the surfaces of the ceramic particles, and a trabecular network between them (Figure 1A,E). New ectopic bone and ceramic particles were separated by microCT, and the total bone volume and ceramics volume were quantified (Figure 1A,B). Both were comparable between groups, showing that ZOL did not increase the volume of new bone during the 14 day period (Figure 1B). In both groups, a dense trabecular network was observed between the ceramic particles (Figure 1E). The trabecular bone was covered with osteoblasts and osteoclasts and contained bone marrow with only few adipocytes. Histomorphometric analyses revealed that the amount of bone and bone marrow were comparable among groups (Figure 1C). As expected, the osteoclast number was reduced in ZOL-containing implants (Figure 1D,E).

### 3.2. Zoledronate Local Mid-Term Effect on Ectopic Bone

**Figure 2:**
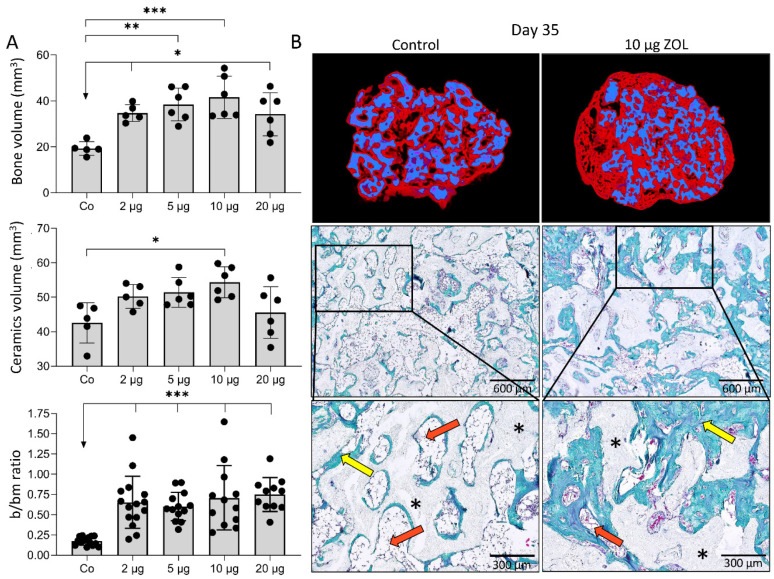
**Different doses of ZOL increased bone volume and affected bone microarchitecture 35 days following subcutaneous implantation of ABGS (rhBMP6/ABC) implants with ceramics.** (**A**) Bone volume (mm^3^) and ceramics volume (mm^3^) quantified by microCT analyses, and histomorphometrically determined bone/bone marrow (b/bm) ratio among control implants (Co) and implants with different doses of ZOL (2, 5, 10 and 20 µg). (**B**) Newly formed bone (red) and ceramics (blue) are shown on 3D reconstruction slices (1st row). Histological sections (Goldner stain) (2nd and 3rd row) of bone-ceramic structure induced by control and implants with 10 µg ZOL. Newly formed bone is marked with yellow arrows, while bone marrow is indicated with orange arrows. Black asterisks indicate ceramic particles. Scale bars are shown in the lower right corner. All *P* values below 0.05 were considered significant and are marked with asterisks as follows: * (*p* ≤ 0.05), ** (*p* ≤ 0.01), *** (*p* ≤ 0.001).

**Figure 3:**
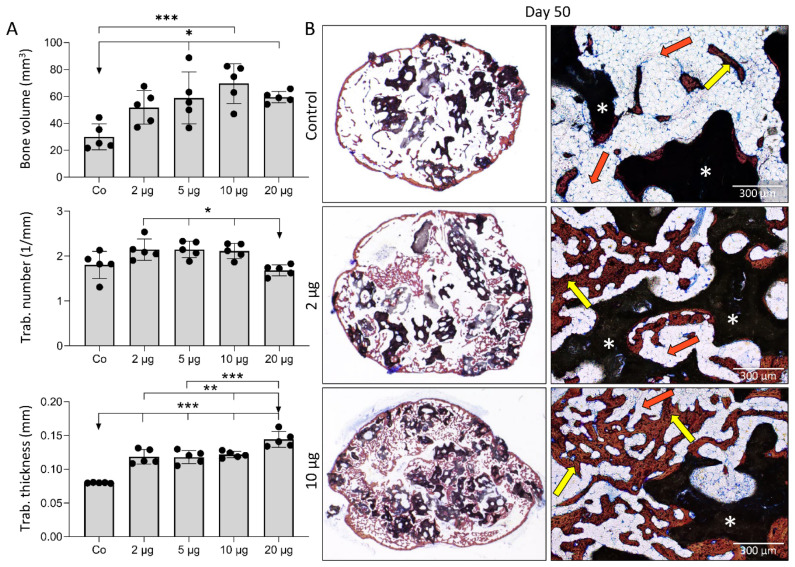
**Different doses of ZOL increased bone volume and affected bone microarchitecture 50 days following subcutaneous implantation of ABGS (rhBMP6/ABC) implants with ceramics.** (**A**) Bone volume (mm^3^), trabecular number (1/mm), and trabecular thickness (mm) among experimental groups. (**B**) Histological sections (Sanderson’s Rapid Bone Stain with Van Gieson picrofuchsin) through BCS induced by control and implants containing ZOL. Newly formed bone is marked with yellow arrows, while bone marrow is indicated with orange arrows. White asterisks indicate ceramic particles. Scale bars are shown in the lower right corner. All *P* values below 0.05 were considered significant and are marked with asterisks as follows: * (*p* ≤ 0.05), ** (*p* ≤ 0.01), *** (*p* ≤ 0.001).

On day 35 and 50 following implantation, new bone was present in all implants (Figure 2 and Figure 3). MicroCT analyses revealed that both on day 35 and day 50, the amount of bone was, in general, significantly higher in implants containing 2–20 µg ZOL compared to controls (Figure 2A and Figure 3A). The bone volume was the highest with 10 µg of ZOL; however, differences between implants containing various ZOL amounts were insignificant. ZOL also decreased the ceramics resorption (Figure 2A), leading to higher ceramics volume in implants containing ZOL than the control implants. The trabecular number was slightly increased in the implants containing 10 µg of ZOL, but the difference was not significant (Figure 3A). The trabecular thickness was higher in implants with ZOL with a dose-dependant effect (Figure 3A).

Analyses of histological and microCT sections revealed that on day 35 and 50 in the implants without ZOL, the bone volume was reduced, and there were only few trabeculae surrounded by bone marrow, with the predominance of adipocytes (Figure 2B and Figure 3B). Therefore, the bone in the control implants mainly consisted of cortical bone and a bone present on the ceramic particle surfaces. On the contrary, implants with ZOL contained a much denser trabecular network (Figure 2B and Figure 3B). In all of the implants, adipocytes were the dominant cells in the bone marrow. However, implants with ZOL had increased bone volume, while the bone marrow area was decreased at day 35 and 50 compared to the control implants (Figure 2B and Figure 3B). The bone/bone marrow ratio was higher in the implants with ZOL (Figure 2A). No remarkable differences existed in the histological and structural features between the implants containing different ZOL doses (Figure 3B).

### 3.3. Local Versus Systemic Zoledronate Effect on Ectopic Bone Volume and Structure

**Figure 4:**
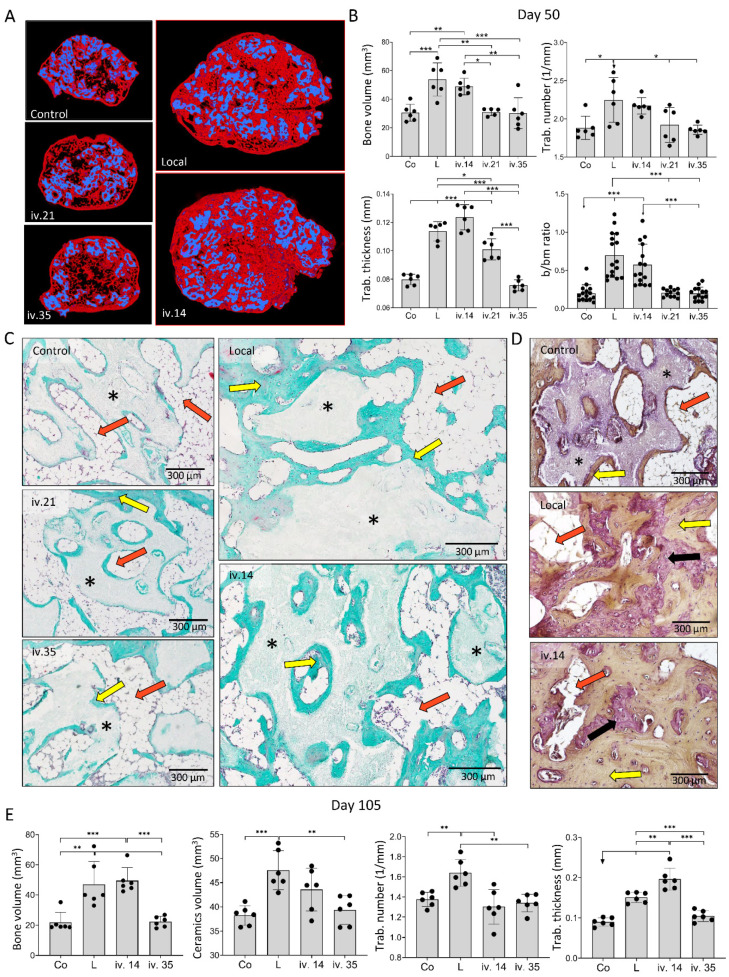
**ZOL applied locally in implant or intravenously (i.v.) on day 14 increased bone volume and affected bone microarchitecture on day 50 and 105 following subcutaneous implantation of ABGS (rhBMP6/ABC) implants with ceramics.** (**A**) Newly formed bone (red) and ceramics (blue) are shown on 3D reconstruction slices on day 50 induced by implants without ZOL (control) or with ZOL applied directly in the implant (local) or intravenously (i.v.) on day 14, 21, or 35. (**B**) MicroCT parameters including bone volume (mm^3^), trabecular number (1/mm), trabecular thickness (mm), and histomorphometrically determined bone/bone marrow (b/bm) ratio on day 50 induced by implants without ZOL (Co) or with ZOL applied directly into the implant (L) or applied intravenously (i.v.). (**C**) Histological sections (Goldner stain) through the bone–ceramic structure (BCS) on day 50. Newly formed bone is marked by yellow arrows, black asterisks indicate ceramic particles, while orange arrows indicate bone marrow. Scale bars are shown in the lower right corner. (**D**) Histological sections (HE stain) through the bone–ceramic structure. Black asterisks indicate ceramic particles, orange arrows indicate bone marrow, black arrows indicate woven bone, while yellow arrows indicate lamellar bone. (**E**) MicroCT parameters including bone volume (mm^3^), ceramics volume (mm^3^), trabecular number (1/mm), and trabecular thickness (mm) among experimental groups on day 105. ZOL was applied locally into the implant (L) i.v. on day 14 or 35. Implants without ZOL served as a control (Co) group. All *P* values below 0.05 were considered significant and are marked with asterisks as follows: * (*p* ≤ 0.05), ** (*p* ≤ 0.01), *** (*p* ≤ 0.001).

**Figure 5:**
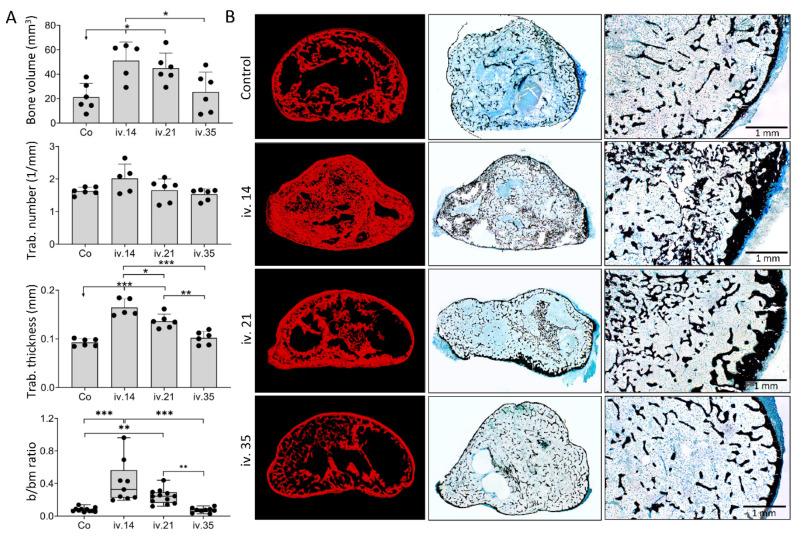
**ZOL applied intravenously on day 14 or 21 increased bone volume and affected bone microarchitecture on day 50 following subcutaneous implantation of ABGS (rhBMP6/ABC) implants without ceramics.** (**A**) MicroCT parameters including bone volume (mm^3^), trabecular number (1/mm), trabecular thickness (mm), and the histomorphometrically determined bone/bone marrow (b/bm) ratio were observed among the experimental groups on day 50. ZOL was applied intravenously (i.v.) on day 14, 21, or 35. Implants without ZOL served as a control (Co) group. (**B**) Newly formed bone (red) is shown on 3D reconstruction slices among experimental groups. Histological (Von Kossa stain) sections through the newly formed bone (stained black) on day 50 induced by implants without ZOL (Co) or with ZOL applied intravenously (i.v.) on day 14, 21, or 35. Scale bars are shown in the lower right corner. All *P* values below 0.05 were considered significant and are marked with asterisks as follows: * (*p* ≤ 0.05), ** (*p* ≤ 0.01), *** (*p* ≤ 0.001).

Bone volume, trabecular number, and trabecular thickness on day 50 and 105 following implantation of ABGS (rhBMP6/ABC) with ceramics were higher in implants with ZOL than those without ZOL when used locally or systemically on day 14, but not when given i.v. on days 21 or 35 following implantation (Figure 4). Moreover, when ZOL was administered i.v. on day 14 or 21 to rats with ABGS implants without ceramics, the bone volume and trabecular thickness were increased as well, while the administration of ZOL on day 35 was similarly ineffective (Figure 5A).

On day 50 and 105 following implantation, the new bone was preserved in implants with different microarchitecture depending on the implant formulation (Figure 4 and Figure 5). ABGS implants containing ceramic particles induced the formation of BCS consisting of the cortical bone forming BCS boundaries, bone present on the surface of ceramic particles, and trabecular bone between ceramic particles (Figure 4C,D) with bone marrow rich in adipocytes. When ZOL was applied locally or i.v. on day 14, the trabecular bone was significantly denser (Figure 4C) and thicker than in implants without ZOL. Moreover, there were areas of compact-like bone between the particles consisting of lamellar and woven bone divided with cement lines (Figure 4D). The bone marrow was predominantly adipocytic but reduced in implants containing ZOL. When ZOL was applied i.v. on day 21, the number and thickness of the trabeculae were increased when compared to controls, but these values were less than those in implants to which ZOL was administered locally or i.v. on day 14 (Figure 4B,E). ZOL administration on day 35 had no influence on the bone volume or the bone marrow area (Figure 4A–C,E).

The new bone induced by ABGS implants without ceramics on day 50 consisted of the cortical bone forming boundaries of the implants and the trabecular bone with bone marrow mainly containing adipocytes (Figure 5B). In the experimental group where ZOL was applied i.v. on day 14, the trabecular number and thickness as well as thickness of the cortical bone increased (Figure 5A,B), while in the group where a single dose of ZOL was applied i.v. on day 21, the effect was less pronounced compared to day 14 (Figure 5A,B). Lastly, in the group where ZOL was applied on day 35 following implantation, no effect on the bone structure and volume was observed (Figure 5A,B).

### 3.4. Maintenance of Ectopic Bone over One Year Follow Up

**Figure 6:**
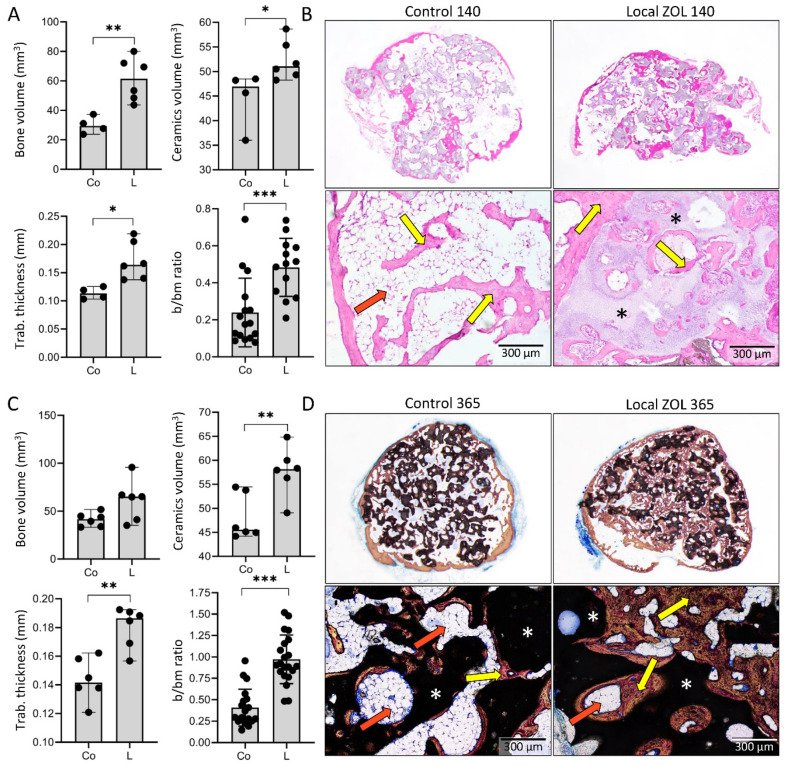
**Effect of ZOL on the amount and microarchitecture of bone–ceramic structure (BCS) is persistent one year following the subcutaneous implantation of ABGS (rhBMP6/ABC) implants with ceramics.** MicroCT parameters including bone volume (mm^3^), ceramics volume (mm^3^), trabecular thickness (mm), and histomorphometrically determined bone/bone marrow (b/bm) ratio on (**A**) day 140 or (**C**) day 365 among control implants (Co) and implants with locally added ZOL (L). Histological sections of BCS on (**B**) day 140 (HE stain) and (**D**) day 365 (Sanderson’s Rapid Bone Stain with Van Gieson picrofuchsin) following subcutaneous implantation of ABGS (rhBMP6/ABC) implants containing ceramics with and without ZOL. Yellow arrows indicate bone, orange arrows indicate bone marrow, while ceramics are indicated with asterisks. Scale bars are shown in the lower right corner. All *P* values below 0.05 were considered significant and are marked with asterisks as follows: * (*p* ≤ 0.05), ** (*p* ≤ 0.01), *** (*p* ≤ 0.001).

The long-term effect of ZOL on the microarchitecture of BCS and the amount of new bone was further analyzed on day 140 and 365 following implantation. At both time points, the bone volume and trabecular number and thickness were higher in implants containing ZOL (Figure 6A,C). Notably, at both time points, the amount of ceramics was higher with ZOL, suggesting that ZOL prevented ceramics resorption (Figure 6A,C).

The microarchitecture of BCS on day 140 and 365 (long-term effect) was not different compared to day 35 and 50 (mid-term effect). BCS induced in control implants (without ZOL) consisted of cortical bone, bone on the ceramic surface, and few trabeculae in between (Figure 6B,D), while the application of ZOL locally resulted in an increased trabecular number and thickness forming a dense network between ceramic particles (Figure 6B,D). Moreover, areas of compact-like bone were maintained, and the bone marrow in all implants was predominantly adipocytic and more extended in implants without ZOL.

## 4. Discussion

**Figure 7:**
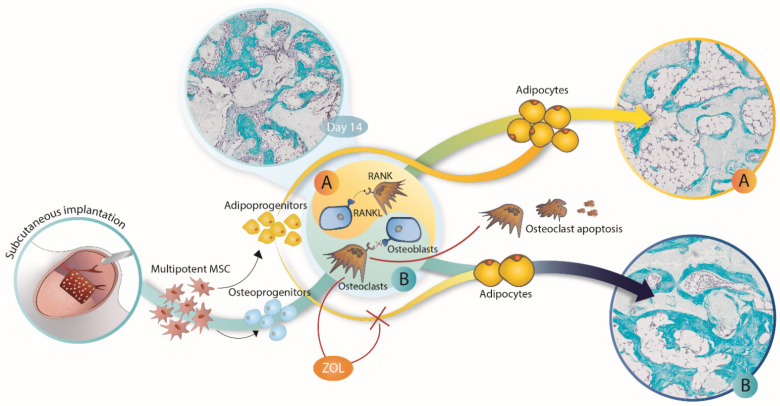
**Zoledronate (ZOL) effect on different stages of BMP-induced ectopic bone formation.** ZOL applied locally into implants (rhBMP6/ABC/ceramics) did not affect the bone volume on day 14 following implantation but decreased the number of osteoclasts. However, the effect of applied ZOL became apparent at later time points when the bone volume in implants without ZOL (**A**) decreased and was accompanied by predominantly adipogenic bone marrow. On the contrary, the addition of ZOL into implants preserved bone volume and significantly decreased adipogenic bone marrow (**B**). The same effect was achieved when ZOL was applied systemically on day 14 but not on day 21 and later, suggesting that adipogenic differentiation at the ectopic site is an irreversible process.

In this study, we have demonstrated that zoledronate bound to ceramics in ABGS implants increased the bone volume at an ectopic subcutaneous site and prevented bone marrow expansion and adipogenesis over a period of 365 days following implantation in rats (Figure 7). ABGS containing rhBMP6 within ABC is a novel biomaterial and has been clinically tested as a therapy for bone regeneration in various orthopaedic and trauma indications [12,13,14]. Moreover, the potential use of ABC and autologous platelet concentrates was also recently evaluated in dental medicine [44,45]. To improve the biomechanical properties of ABGS, different ceramics and allograft formulations were used as CRM [14,15,17]. The geometry and size of synthetic ceramic particles determine the microarchitecture of newly formed bone, thus being a powerful tool in bone tissue engineering [17]. Bone induced by ABGS implants with ceramics in a rat subcutaneous assay reached its peak volume on day 14 followed by bone volume decreases and adipogenic differentiation of the bone marrow at later time points [18].

The addition of ZOL in implants significantly affected bone quantity and structure at different time points of ectopic osteogenesis within a one-year follow-up in rats. ZOL increased both the bone volume and the amount of ceramics at all prolonged observation time points. This is in line with previously published studies in which collagen or ceramics (calcium phosphate or calcium sulfate) were used as a BMP carrier in rat ectopic or orthotopic models [29,30,31,32,34,35,36,37]. However, we show here for the first time that ZOL affects bone volume and morphology over a period of one year following subcutaneous or systemic use, which comprises about 50% of the rat’s life expectancy.

Although the dense bone structure observed in ABGS implants with ZOL was composed of lamellar and woven bone, it was compact and fused in a solid bone structure with a long survival period. The abundance of bone, regardless of its woven/immature nature, might contribute to more resistant ectopic bone biomechanical support to prevent nerve compression after spinal fusion in patients with lumbar back pain. The increased longevity of bone at ectopic sites in rats suggests that the administration of bisphosphonates with ABGS and ceramics might be a promising solution for their local use to enhance ectopic bone volume and longevity in support of the functional outcome of human spine surgeries and eventually large bone defect repair. The previous demonstration that the co-application of bisphosphonates with BMPs improved the biomechanical properties of calvarial bone implants [34] suggests that the addition of bisphosphonates into ABGS might increase biomechanical bone properties.

In addition, ZOL increased the amount of bone and decreased the area containing bone marrow, specifically the number of adipocytes, resulting in a significantly increased bone/bone marrow ratio in a dose-independent manner, leading to more dense and compact bone tissue.

BMPs induce the osteogenic and chondrogenic differentiation of MSCs but also support their adipogenic differentiation [46]. On the other hand, it is well known that bisphosphonates have an anti-catabolic effect in bone via inhibiting the function and by reducing the number of osteoclasts and bone resorption [28,47]. Moreover, it was also demonstrated *in vitro* that bisphosphonates (alendronate) decrease the expression of adipogenic transcription factors such as peroxisome proliferator activated receptor gamma 2 (PPARγ2), thus inhibiting the adipogenic differentiation of MSCs [48,49]. Alendronate might eventually stimulate the differentiation of osteoblasts from mesenchymal stem cells [48,49]. However, in this study, ZOL did not affect the bone volume at day 14. This implies that ZOL did not affect the bone induction/formation and that the ZOL effect on the implant bone quantity and structure at later time points was related to the inhibition of bone resorption.

Notably, the findings indicated that the ZOL effect was both administration- and time-dependent. The bone volume was equivalently increased if it was applied locally at the time of implantation or intravenously on day 14 following the implantation of ABGS implants with ceramics. However, if applied later, such as on day 35, the bone volume compared to control implants was unchanged. In the case of the ABGS implants without ceramics, ZOL applied intravenously on day 14 or 21 achieved the optimal effect, while administered later, such as on day 35, the bone volume was equivalent to that of the control implants. These studies showed that the equivalent effect of bisphosphonates on newly induced bone was achieved both by local and systemic administration at indicated time points during bone formation. Locally administered ZOL in implants is preferred to systemic administration because it will eventually avoid side effects attributed to the systemic use of bisphosphonates like in patients with osteoporosis, including osteonecrosis of the jaw, atrial fibrillation, atypical femoral fractures, and other [50,51,52,53]. The dose of 20 µg ZOL administered locally into the ABGS implant with synthetic ceramics or systemically (i.v.) to those with or without ceramics was equal to 0.06 mg ZOL/kg of rat body weight. This was close to the i.v. dose of 4 mg ZOL given to humans (70 kg body weight) at a ratio of around 0.05 mg/kg. Therefore, the release of ZOL from the ceramics is possible, and this could effect the skeleton. Since ZOL affected the implant bone volume when given systemically, its effects on the entire skeleton were likely to occur and had an extended positive outcome. However, the local use of ZOL in combination with ABGS for bone repair, including posterolateral spinal fusion, resistant atrophic non-unions and, congenital pseudoarthoris of the tibia in children, could be used as an option to increase the bone volume and long-term survival at the ectopic and orthotopic skeletal site.

## 5. Conclusions

When used in ABGS implants with ceramics, ZOL increased the bone and ceramics volume by decreasing resorption. The effect of ZOL was equivalent when ZOL was applied either locally into the implant or intravenously on day 14 after implantation but not if injected on day 35. Importantly, the effect of ZOL on the amount and structure of the bone was maintained over a period of one year following implantation.

The results presented here might be useful in spine bone fusion indications when new ectopic bone is induced between adjacent transverse processes or between vertebral bodies and when it is maintained for a long time period in patients with degenerative disc disease, scoliosis, and spondylolisthesis.

## Data Availability

Raw data were generated at the Laboratory for Mineralized Tissues. Derived data supporting the findings of this study are available from the corresponding author, S.V., upon request.

## Abbreviations

BMPs	bone morphogenetic proteins
ABC	autologous blood coagulum
ABGS	autologous bone graft substitute containing rhBMP6 within autologous blood coagulum
BCS	Bone–ceramic structure
ZOL	zolendronate
TCP/HA	beta-tricalcium phosphate/hydroxyapatite
